# HIF1A overexpression predicts the high lymph node metastasis risk and indicates a poor prognosis in papillary thyroid cancer

**DOI:** 10.1016/j.heliyon.2023.e14714

**Published:** 2023-03-17

**Authors:** Yong-xun Zhao, Ze Yang, Li-bin Ma, Fang Wang, Yong Wang, Cheng Xiang

**Affiliations:** a The Seventh Department of General Surgery, The First Hospital of Lanzhou University, Lanzhou, 730000, Gansu, China; b The Pathology Department, The First Hospital of Lanzhou University, Lanzhou, 730000, Gansu, China; c Department of Thyroid Surgery, The Second Affiliated Hospital of Zhejiang University School of Medicine, Hangzhou, 310009, Zhejiang, China

**Keywords:** HIF1A expression, Papillary thyroid cancer, Lymph node metastasis, Prognosis

## Abstract

**Objective:**

To investigate the value of Hypoxia-inducible factor 1 A (HIF1A) in predicting lymph node metastasis (LNM) stage and clinical outcomes of papillary thyroid cancer (PTC) patients.

**Materials and methods:**

The HIF1A gene expression analysis in PTC was performed by bioinformatics approaches followed by evaluating its protein level using immunohistochemistry analysis. The role of HIF1A in predicting the LNM stage was evaluated by logistic regression analysis, nomogram construction, and receiver operating characteristic (ROC) analysis. We performed survival analyses to determine its prognostic value. Enrichment analysis was conducted, and immune cell infiltration and stromal content were evaluated to examine the underlying mechanism of HIF1A in PTC.

**Results:**

HIF1A transcription and protein levels were significantly high in PTC tissue (P < 0.05). Its overexpression predicted high LNM risk and unfavorable prognosis for PTC patients (P < 0.05). Cox regression analysis revealed HIF1A as an independent prognostic biomarker for the disease-free interval (DFI) (P < 0.01). In addition, HIF1A was positively related to tumor-suppressive immunity but was negatively correlated with anti-tumor immunity. HIF1A upregulation was also associated with increased stromal content.

**Conclusions:**

HIF1A overexpression is an independent predictor for worse DFI in PTC. The HIF1A expression may affect the prognosis of PTC patients through immune- and stroma-related pathways. Our study provides new insight into the role of HIF1A in PTC biology and clinical management.

## 1 Introduction

Thyroid cancer is the most common endocrine malignancy with an increasing incidence globally in recent years. Thyroid cancer ranked fourth in the number of all estimated new cases of cancers in 2021, while it was ranked fifth and sixth in 2020 and 2019, respectively [[1], [2], [3]]. It includes papillary, follicular, medullary, and undifferentiated types [4]. Papillary thyroid cancer (PTC) is the most prevalent histological type of carcinoma with an 80% prevalence [5]. PTC patients in early-stage have an overall 5-year survival of over 90% using established methods [6]. Although most PTC patients have a favorable prognosis, a small proportion of patients still suffer from local invasion or distant metastasis, leading to a low survival time [7]. Lymph node metastasis (LNM) occurs in 14%–64% of PTC patients and is associated with recurrence [8]. Besides, the enhanced angiogenesis pathway also results in the progression of PTC [9]. Therefore, it is urgent to find novel biomarkers for predicting the LNM stage and survival to improve the prognosis of PTC patients.

Hypoxia-inducible factor 1 (HIF-1) contains an oxygen-regulated subunit (HIF1A) and a constitutively expressed subunit [10]. Upon hypoxia, HIF-1 binds to the hypoxia response elements (HREs), regulating multiple pathological and physiological processes including apoptosis, glucose metabolism, and angiogenesis [11]. Specifically, HIF1A participates in adapting cancer cells to lower oxygen levels and it may be a late recurrence biomarker [12]. The activation of HIF1A promotes the Warburg effect by switching from oxidative phosphorylation to glycolysis [11]. HIF1A expression is low in most cells under normal conditions, but HIF1A is often significantly elevated under hypoxia [13]. HIF1A is overexpressed in cancers with involvement in biological processes of tumor cell survival, angiogenesis, metastasis, and tumor therapy [14,15]. Besides, HIF1A is expressed in lymphocytes, dendritic cells, neutrophils, and macrophages, thereby regulating the immune microenvironment [16]. The previous study has demonstrated upregulated HIF1A expression in breast tumors compared with normal breasts and served as a potential prognostic biomarker for cancer recurrence [12]. However, there is no systemic research on the expression, clinical significance, and underlying mechanism of HIF1A in PTC.

This study aimed to find a potent biomarker to predict LNM stage and prognosis in PTC. In the area of big data, bioinformatics as a powerful tool for data mining was adopted for HIF1A expression analysis. We also examined value of HIF1A in predicting LNM stage and prognosis of PTC patients. The enrichment analysis, evaluation of immune cell infiltration, and stromal content were employed to elucidate the underlying mechanism of HIF1A in PTC.

## 2 Materials and Methods

### 2.1 Data acquisition

Due to the small number of normal thyroid samples in the TCGA database, the data from TCGA combined with GTEx (including 504 thyroid cancer samples and 338 normal thyroid samples) were obtained. The transcriptome profiles (HTSeq-FPKM), phenotype information, and survival data were downloaded from the GDC TCGA sets in the UCSC Xena database (https://xenabrowser.net/). Inclusion criteria: PTC samples; patients had complete survival and expression information. At last, we included 497 PTC samples for following analyses, and they were divided into low- and high-HIF1A expression groups according to the median HIF1A expression. The patient characteristics were shown in Table S1. Besides, as the following inclusion criteria: >100 samples; containing thyroid cancer and normal samples; with expression data, the GSE33630 dataset (containing 59 tumorous and 46 normal samples) was generated to verify HIF1A expression.

### 2.2 Expression analysis of HIF1A

TIMER (http://timer.comp-genomics.org/) was used to explore the differential HIF1A mRNA level in various cancers. Then, HIF1A gene expression in PTC was analyzed using the GDC TCGA together with GTEx data, validated by the GSE33630 dataset. Following the manufacturer's protocol, we carried out immunohistochemistry (IHC) analysis for acquiring its protein expression.

Next, the relationship between HIF1A levels and clinical parameters of PTC patients including age, gender, LNM stage, and subtype was evaluated using the chi-square test, and univariate logistic regression analysis. Female was set as a reference level for gender, N0 stage for the LNM stage, and PTC-classical for the histological subtype.

### 2.3 The clinical significance of HIF1A

Firstly, the association of the LNM stage with HIF1A expression and the other clinical parameters was determined using logistic regression analysis. After that, we constructed a nomogram. Besides, we drew the ROC curves to examine the value of the HIF1A together with clinical factors in predicting the LNM stage.

Following this, the effect of HIF1A expression on survival was analyzed using the Kaplan-Meier plotter method. The survival differences between two expression groups were compared by the log-rank test. Further, the independent predictors for PTC were identified using Cox regression analysis.

### 2.4 Functional enrichment analysis

The limma package was employed for differential expression analysis between the two HIF1A groups. The significant differentially expressed genes (DEGs) were identified by setting |log 2 foldchange |>1 and P < 0.05 as the selection criterion. Gene ontology annotations and possible pathways enriched by the significant DEGs were analyzed with clusterProfiler package [17].

### 2.5 Gene set enrichment analysis (GSEA)

To further explore the biological signaling pathways that HIF1A was involved in, GSEA was executed between two HIF1A expression groups. The gene set was permutated 1000 times and HIF1A expression level was used as a phenotypic label.

### 2.6 Single-sample gene-set enrichment analysis (ssGSEA)

Moreover, we defined the enrichment level of pathways in PTC samples as the ssGSEA score [18]. The gene set represents the collection of all marker genes of a pathway.

### 2.7 Evaluation of the immune cell infiltration and stromal content

CIBERSORT algorithm is a tool for estimating the abundances of 22 immune cells using transcriptomic data [19,20]. We used this algorithm to calculate relative immune cell proportions in two HIF1A expression groups. In addition, ESTIMATE algorithm was used to calculate the Immune Score (represents the infiltration of immune cells in tumor tissue), the Stromal Score (captures the presence of stroma in tumor tissue), and the ESTIMATE Score (equaled the sum of Stromal Score and Immune Score) [21].

### 2.8 Statistical analysis

SPSS software (version 23.0) and R software (version 4.0.3) were used for all statistical analyses. Differences in each two-group comparison and among at least three groups were determined by a *t-test* and one-way ANOVA, respectively. Pearson's correlation test was adopted for correlation analysis. P < 0.05 was considered statistically significant.

## 3 Results

### 3.1 HIF1A expression is increased in PTC patients

**Fig. 1:**
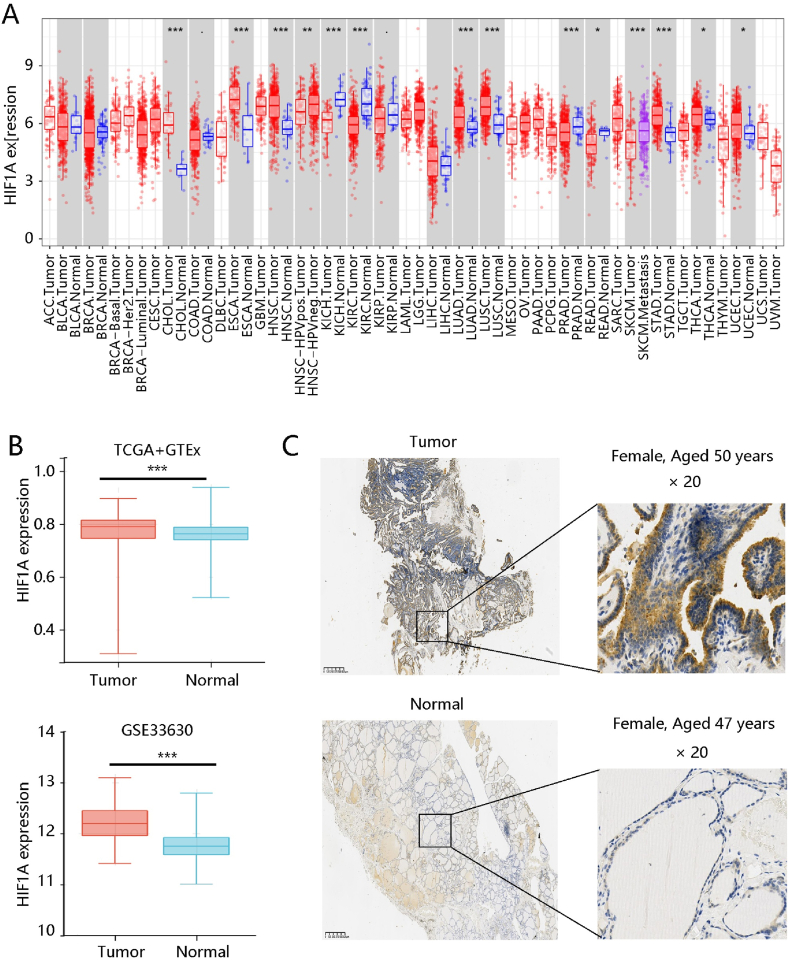
Higher HIF1A expression in papillary thyroid cancer (PTC) patients. **(A)** The HIF1A mRNA expression in pan-cancer. **(B)** The obviously higher HIF1A mRNA level in PTC than that in normal group using the TCGA combined with GTEx data, validated by the GSE33630 dataset. **(C)** The higher protein level of HIF1A in PTC tissue using immunohistochemistry analysis. *P < 0.05, ***P < 0.001.

The HIF1A mRNA level in human cancers was visualized in Fig. 1A. Then, we found that HIF1A gene was elevated in PTC compared to normal thyroid (P < 0.001). GSE33630 data further confirmed the HIF1A overexpression in the tumor tissue (P < 0.001) (Fig. 1B). IHC staining exhibited that HIF1A protein was obviously upregulated in PTC (Fig. 1C). These results indicated high HIF1A expression in PTC.

### 3.2 HIF1A is linked to LNM stage and subtype

We analyzed the distribution of different clinicopathological parameters in two HIF1A groups. There was no significantly different distribution of age and gender (P > 0.05), while the N1 stage was mainly distributed in the high HIF1A group (P < 0.001). Besides, the histological subtype was significantly related to HIF1A expression (P < 0.001) (Table 1).

**Table 1:** Relationship between HIF1A levels and the clinicopathological parameters of PTC patients in GDC TCGA.

Variables	Total	Low HIF1A	High HIF1A	χ^*2*^	*P*-value
	n = 497 (%)	n = 248 (%)	n = 249 (%)		
Age (years)				0.365	0.546
<55	333 (67.0)	163 (65.7)	170 (68.3)		
≥55	164 (33.0)	85 (34.3)	79 (31.7)		
Gender				0.186	0.666
Female	363 (73.0)	179 (72.2)	184 (73.9)		
Male	134 (27.0)	69 (27.8)	65 (26.1)		
LNM stage				16.117	<0.001
N0	224 (45.1)	130 (52.4)	94 (37.8)		
N1	225 (45.3)	90 (36.3)	135 (54.2)		
Unknown	48 (9.6)	28 (11.3)	20 (8.0)		
Subtype				72.201	<0.001
PTC-classical	358 (72.0)	151 (60.9)	207 (83.1)		
PTC-tall cell	37 (7.4)	9 (3.6)	28 (11.2)		
PTC-follicular	102 (20.5)	88 (35.5)	14 (5.6)		

**Abbreviations:** LNM stage, lymph node metastasis; PTC, papillary thyroid cancer.

**Fig. 2:**
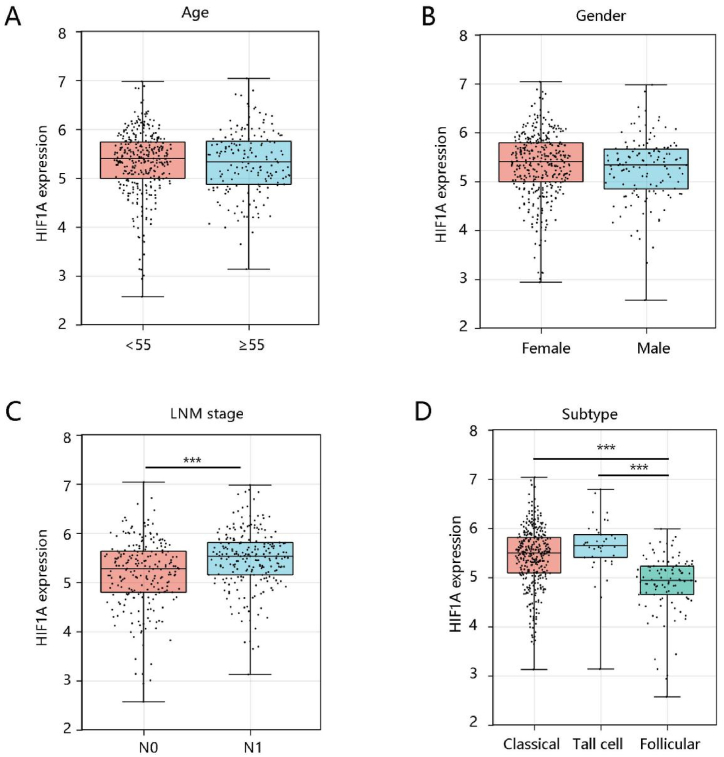
The association of HIF1A with clinical features. **(A)** Age. **(B)** Gender. **(C)** LNM stage. **(D)** Subtype. **Abbreviations:** LNM stage, lymph node metastasis.

When considering HIF1A as a continuous variable, HIF1A was not associated with age and gender as well (Fig. 2A–B). However, HIF1A was significantly linked to LNM stage and subtype (P < 0.001) (Fig. 2C–D). As expected, patients at the N1 stage had higher HIF1A expression compared with those at the N0 stage (P < 0.05) (Fig. 2C). Additionally, PTC-tall cell had the highest HIF1A expression, followed by PTC-classical, and PTC-follicular (Fig. 2D).

Through the logistic regression analysis, age and gender had no remarkable correlation with the HIF1A expression. The high LNM stage was associated with HIF1A overexpression with an odds ratio (OR) of 2.071 (P < 0.001). Besides, PTC-tall cell and PTC-follicular were also significantly correlated with HIF1A expression (P < 0.05) (Table 2).

**Table 2:** The correlation between HIF1A expression and clinical features using univariate logistic regression analysis.

Characteristics	Odds ratio	95% confidence interval	*P*-value
Age	0.996	0.985–1.007	0.453
Gender	0.916	0.617–1.362	0.666
LNM stage	2.074	1.424–3.022	<0.001
PTC-tall cell	2.269	1.041–4.950	0.039
PTC-follicular	0.116	0.064–0.212	<0.001

Abbreviations: LNM stage, lymph node metastasis; PTC, papillary thyroid cancer.

### 3.3 HIF1A overexpression predicted the high LNM risk

**Fig. 3:**
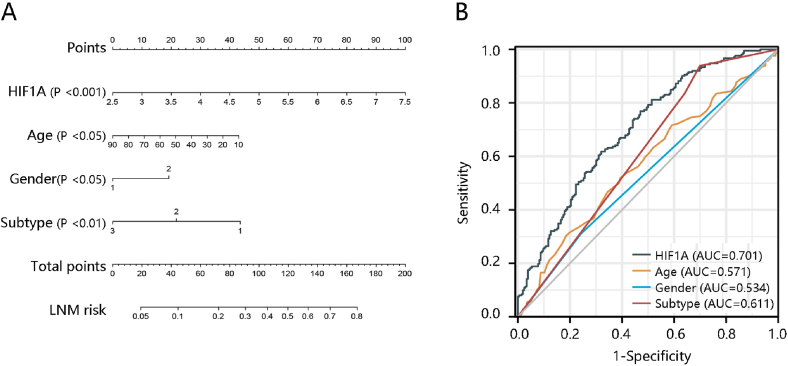
HIF1A overexpression predicted high lymph node metastasis (LNM) risk. **(A)** A nomogram was constructed. **(B)** Receiver operating characteristic curve analysis revealed the superior performance of HIF1A in predicting LNM risk. **Note:** patients at N1 stage (n = 225), patients at N0 stage (n = 224).

Due to the significant relation between HIF1A expression and the LNM stage, and the prognostic value of the LNM stage in PTC [22], we further analyzed its value in predicting LNM stage. HIF1A, age, gender, and subtype could independently predict the LNM stage (P < 0.05). Of note, HIF1A overexpression was connected with high LNM stage risk with an OR of 1.796 (P < 0.001) (Table 3). After integrating independent factors into nomogram construction, HIF1A contributed the most to the LNM risk (Fig. 3A). Moreover, the AUC of the ROC curve of HIF1A was 0.701, which was superior to age (AUC = 0.571), gender (AUC = 0.534), and subtype (AUC = 0.611), indicating that HIF1A presented an acceptable performance in predicting the LNM stage (Fig. 3B).

**Table 3:** The association of the LNM stage with the HIF1A expression and clinicopathological parameters using logistic regression analysis.

Characteristics	Univariate logistic regression		Multivariate logistic regression	
	OR (96% CI)	*P*-value	OR (96% CI)	*P*-value
HIF1A	2.074 (1.524–2.824)	<0.001	1.796 (1.299–2.482)	<0.001
Age	0.986 (0.975–0.998)	0.024	0.984 (0.972–0.997)	0.016
Gender	1.525 (1.003–2.318)	0.048	1.753 (1.116–2.754)	0.015
Subtype	0.470 (0.357–0.617)	<0.001	0.529 (0.397–0.704)	<0.001

**Abbreviations:** OR, odds ratio; 95% CI, 95% confidence interval.

**Note:** patients at N1 stage (n = 225), patients at N0 stage (n = 224).

### 3.4 High HIF1A mRNA expression leads to poor prognosis

**Fig. 4:**
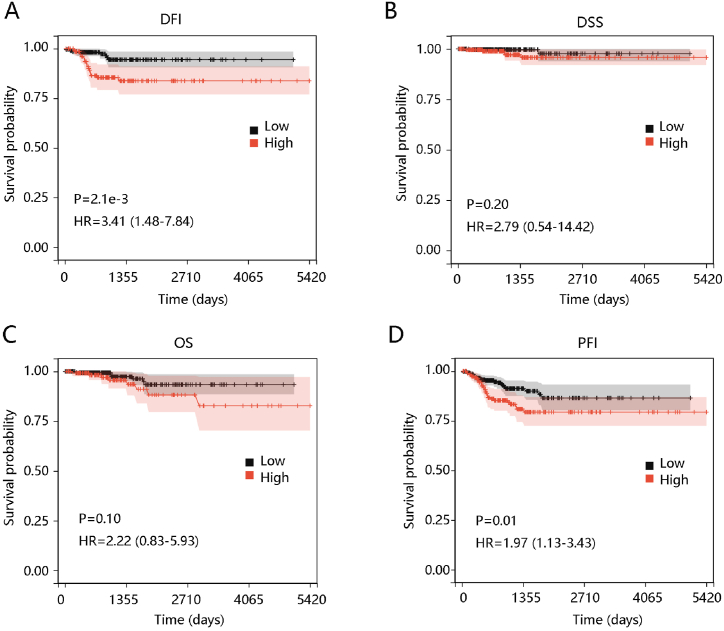
Effect of HIF1A expression on patient prognosis. (A) Disease-free interval (DFI). (B) Disease-specific survival (DSS). (C) Overall survival (OS). (D) Progression-free interval (PFI).

We plotted the survival curves to explore the prognostic significance of HIF1A in PTC. Fig. 4A–D shows that patients with high HIF1A expression tended to have a shorter DFI and PFI, but had no significant difference in DSS and OS. The stratified analysis presented that HIF1A had a significant impact on DFI and PFI in PTC patients aged below 55 years, females, and the PTC-classical subtype (P < 0.05). In addition, HIF1A had a close relation with DFI in those at N1 status but significantly affected PFI in those at N0 status (P < 0.05) (Table 4).

**Table 4:** The correlation between HIF1A expression and prognosis in PTC patients with different subgroups.

Clinicopathological characteristics	Disease-free interval		Progression-free interval	
	HR (95% CI)	*P*-value	Hazard ratio (95% CI)	P value
Age				
<55	5.03 (1.67–15.15)	1.4e-3	2.36 (1.07–5.20)	0.03
≥55	1.98 (0.44–8.89)	0.36	1.91 (0.86–4.26)	0.11
Gender				
Female	5.12 (1.70–15.43)	1.2e-3	2.73 (1.29–5.76)	6.2e-3
Male	2.73 (0.61–12.20)	0.17	0.50 (0.20–1.25)	0.13
LNM stage				
N0	2.26 (0.50–10.12)	0.27	3.95	6.9e-3
N1	4.17 (1.19–14.65)	0.02	1.71 (0.82–3.55)	0.15
Subtype				
PTC-classical	3.72 (1.36–10.17)	5.9e-3	2.03 (1.04–3.95)	0.03
PTC-tall cell	325,289,474 (0, Inf)	0.35	0.21 (0.02–1.78)	0.12
PTC-follicular	1.7e-9 (0, Inf)	0.09	0.22 (0.04–1.30)	0.07

**Abbreviations:** HR, hazards ratio; 95% CI, 95% confidence interval; LNM, lymph node metastasis; PTC, papillary thyroid cancer.

To identify independent risk factors for DFI and PFI, Cox regression analysis was conducted. In the univariate analysis, the LNM stage and HIF1A were significantly related to DFI and PFI (P < 0.05). Next, these two significant factors were included in the multivariate analysis. They could independently predict DFI but not PFI (Table 5).

**Table 5:** Cox regression analysis of disease-free interval and progression-free interval in PTC patients.

Variables	Univariate analysis		Multivariate analysis	
	HR (95% CI)	P value	HR (95% CI)	P value
**Disease-free interval**				
Age	0.984 (0.957–1.011)	0.247	/	/
Gender	1.201 (0.473–3.047)	0.700	/	/
LNM stage	4.181 (1.519–11.506)	0.006	3.028 (1.087–8.434)	0.034
Subtype	0.630 (0.333–1.190)	0.154	/	/
HIF1A	6.554 (2.206–19.468)	0.001	5.691 (1.704–19.008)	0.005
**Progression-free interval**				
Age	1.017 (0.999–1.035)	0.072	/	/
Gender	1.625 (0.896–2.948)	0.110	/	/
LNM stage	1.925 (1.0128–3.605)	0.041	1.368 (0.784–2.386)	0.270
Subtype	0.747 (0.496–1.126)	0.163	/	/
HIF1A	1.898 (1.070–3.366)	0.028	1.864 (0.990–3.510)	0.054

**Abbreviations:** HR, hazard ratio; 95% CI, 95% confidence interval; LNM, lymph node metastasis; PTC, papillary thyroid cancer.

### 3.5 HIF1A participates in immune- and stromal-associated pathways

**Fig. 5:**
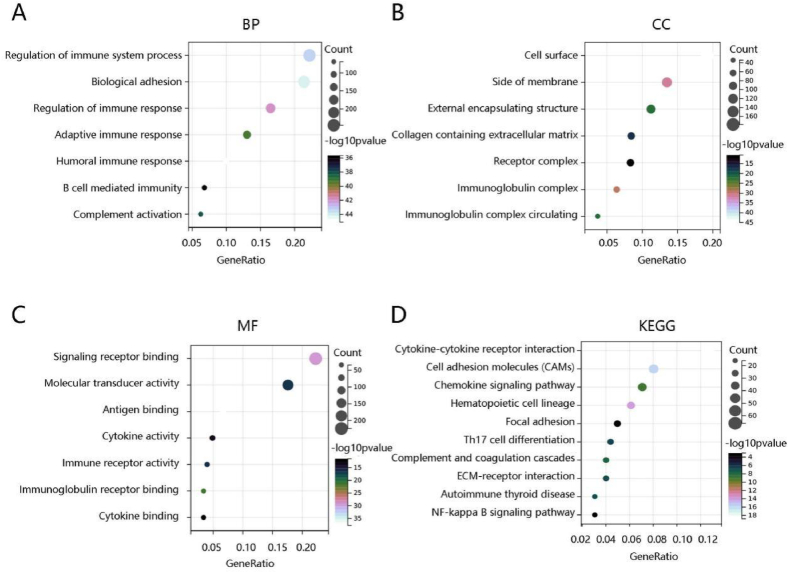
Functional enrichment analysis of significant differentially expressed genes (DEGs) between high- and low- HIF1A expression groups. The **(A)** biological process (BP), **(B)** cellular component (CC), and **(C)** molecular function (MF) of these DEGs. **(D)** KEGG pathway enrichment of the DEGs.

To elucidate biological function of HIF1A for PTC, we first screened 1062 downregulated and 764 upregulated significant DEGs between two expression groups. Then, these DEGs were used for functional enrichment analysis to explore the HIF1A-related pathways. The top 21 significant terms of BP, CC, and MF enrichment analysis are presented in Fig. 5A–C. Notably, in terms of BP, HIF1A was mainly enriched in the regulation of the immune system process, biological adhesion, regulation of immune response, adaptive immune response, humoral immune response, B cell-mediated immunity, and complement activation. Additionally, the major KEGG pathways in which they were involved were cytokine-cytokine receptor interaction, cell adhesion molecules, hematopoietic cell lineage, focal adhesion, ECM-receptor interaction, and autoimmune thyroid disease (Fig. 5D).

**Fig. 6:**
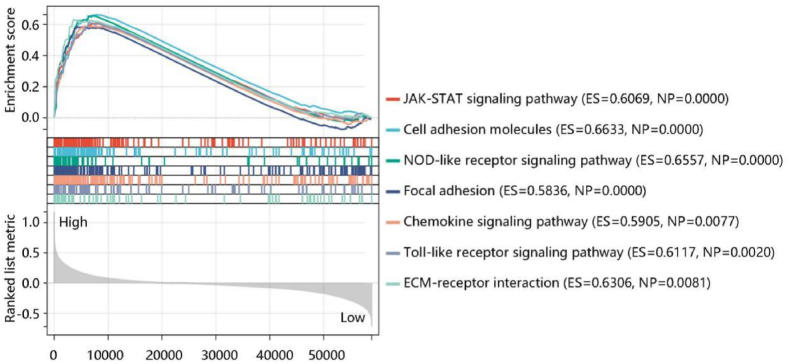
Gene set enrichment analysis revealed the top seven pathways enriched in the high HIF1A expression group. **Abbreviations:** ES, enrichment score; NP, nominal *P*-value.

To further explore the molecular mechanisms affected by HIF1A in PTC, GSEA was executed using GDC TCGA data. JAK-STAT signaling pathway, cell adhesion molecules, NOD-like receptor signaling pathway, focal adhesion, chemokine signaling pathway, Toll-like receptor signaling pathway, and ECM-receptor interaction were enriched in the high HIF1A expression group (Fig. 6). The combined results uncovered that hematopoietic cell lineage, JAK-STAT signaling pathway, Toll-like receptor signaling pathway, cell adhesion molecules, focal adhesion, and ECM-receptor interaction might be important pathways involved in PTC.

**Fig. 7:**
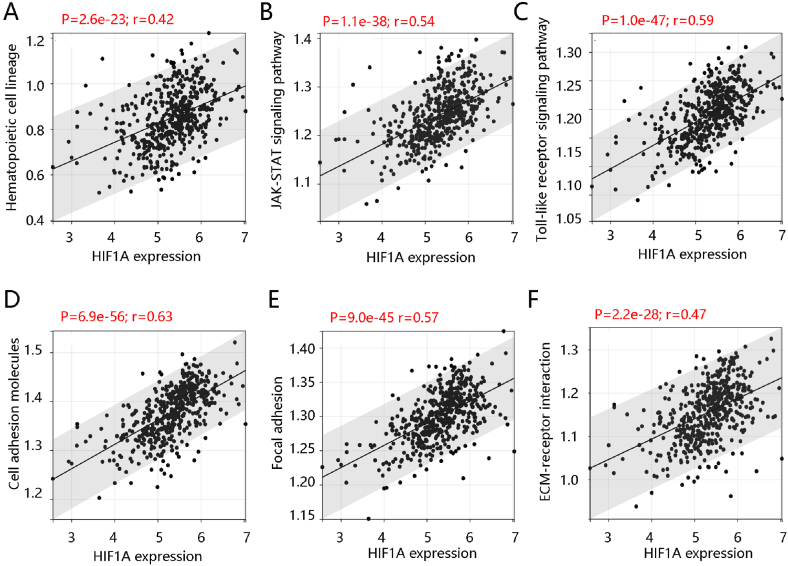
The significantly positive association of HIF1A with the enrichment levels of the six pathways. Pathways of **(A)** Hematopoietic cell lineage, **(B)** JAK-STAT, **(C)** Toll-like receptor, **(D**) cell adhesion, **(E)** focal adhesion, and **(F)** ECM-receptor.

Following this, the enrichment levels of hematopoietic cell lineage, JAK-STAT signaling pathway, Toll-like receptor signaling pathway, cell adhesion molecules, focal adhesion, and ECM-receptor interaction were analyzed using the ssGSEA. The HIF1A gene had a significantly positive relationship with the enrichment levels of the six pathways (all P < 0.001) (Fig. 7A–F). Therefore, HIF1A might affect the PTC progression mainly through positive regulation of immune-related pathways including hematopoietic cell lineage, JAK-STAT, and Toll-like receptor, and by stromal-associated pathways including cell adhesion molecules, focal adhesion, and ECM-receptor interaction.

### 3.6 Association of HIF1A with immune cell infiltration and stromal content

**Fig. 8:**
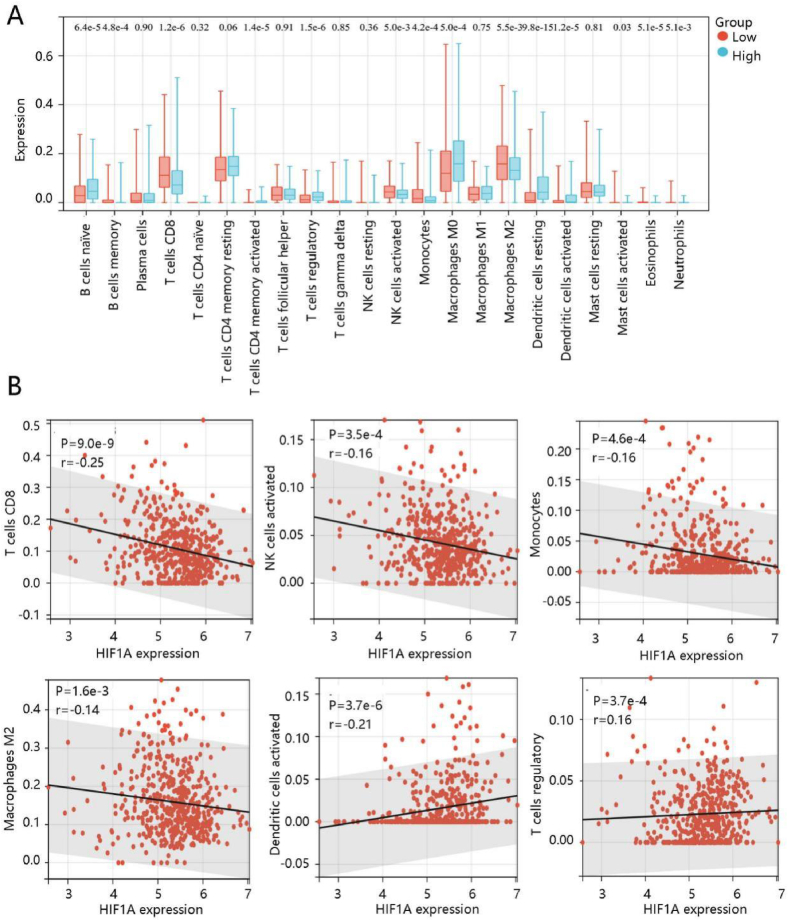
Immune cell infiltration analysis by CIBERSORT. **(A)** The different abundance of 22 immune cells between high- and low- HIF1A expression groups. **(B)** The significant association of HIF1A mRNA expression with T cells CD8, NK cells activated, monocytes, macrophages M2, dendritic cells activated, and T cells regulatory.

We have demonstrated that HIF1A was involved in pathways linked to the immune and stroma; hence, we evaluated the immune cell infiltration and stromal content. The abundance of most immune cells was significantly different between high- and low- HIF1A expression groups (Fig. 8A). HIF1A was negatively correlated with T cells CD8, NK cells activated, monocytes, and macrophages M2, but positively associated with dendritic cells activated, and T cells regulatory (Fig. 8B). These results indicated that HIF1A expression exhibited negative correlations with anti-tumor immunity, and presented a positive association with tumor-suppressive immunity.

**Fig. 9:**
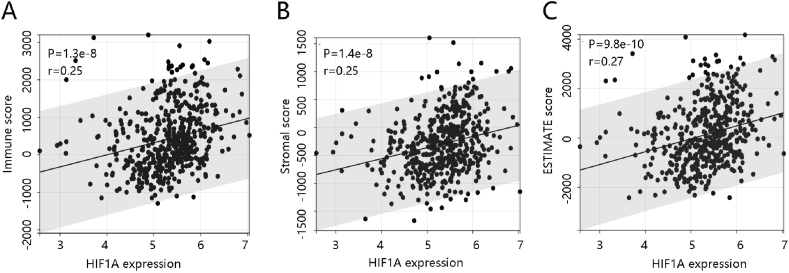
Immune cell infiltration analysis by ESTIMATE. The significant positive correlation between HIF1A and the scores of **(A)** immune, **(B)** stroma, and **(C)** ESTIMATE.

Subsequently, ESTIMATE algorithm was used for evaluation of stromal content. HIF1A was positively related to the immune score, stromal score, and ESTIMATE score (Fig. 9A–C); indicating that the elevated HIF1A level was linked to a more abundant stromal content in cancer, another potential mechanism by which HIF1A regulated PTC progression.

## 4 Discussion

PTC is the most common subtype of thyroid cancer, and patients with PTC always exhibit a good prognosis with radical surgery and radiation therapy; nevertheless, some patients still have aggressive disease and can develop distant metastasis [23]. ANXA10 has been proven to be upregulated in PTC tissue, and its high expression independently predicted the poor prognosis for PTC patients [24]. CXCL10 was a potential core gene of the PTC microenvironment and was significantly associated with prognosis and immune cells in PTC [25]. Despite the rapid progress in PTC biomarkers, the OS rate of PTC patients has not significantly improved in the past 10 years [26]. Our study demonstrates the role of HIF1A in PTC and reveals its associated regulatory pathways by performing a comprehensive analysis of open-access databases.

Hypoxia is a common condition found in a range of solid tumors, which plays an essential role in tumor occurrence and development [27]. Tumor cells adapt to a hypoxic environment mainly regulated by HIF-1 [28]. As a subunit of HIF-A, HIF1A has been implicated as a tumor promoter gene, which was overexpressed in tumors of the ovarian, bladder, and prostate [[29], [30], [31]]. Moreover, HIF1A expression was highly expressed in thyroid cancer compared with that in normal thyroid [32]. However, there is a lack of systemic research about the expression, clinical significance, and underlying mechanism of HIF1A in PTC. Our study also showed an upregulation of HIF1A in PTC tissues compared with the normal thyroid tissue, which was validated by IHC analysis. Besides, HIF1A expression was significantly higher in PTC patients at the N1 stage, and the HIF1A overexpression was significantly related to high LNM risk with an OR = 1.796, which was similar to the result of Liu's study [33]. Moreover, we found that HIF1A was superior to other clinical factors in predicting the LNM stage. It has been demonstrated that HIF1A upregulates epithelial-mesenchymal transition-related transcription factors [34]. These results suggested that HIF1A might represent a crucial role in the invasion, metastasis, and progression of PTC.

The prognostic value of HIF1A in PTC was also assessed using GDC TCGA data. Patients with high HIF1A expression had shorter DFI and PFI but had no significant difference in DSS or OS compared to those with low HIF1A expression. Further, Cox regression analysis revealed HIF1A as a prognostic factor for poor DFI. We have demonstrated that patients with high HIF1A expression are more likely to develop lymph node metastases, which may explain the unfavorable prognosis in the high HIF1A expression group. HIF1A binds to the hypoxia response elements in the promoter region of target genes and is mainly involved in adaptive changes, enabling tumor cells to survive and proliferate in a hypoxic environment, thereby promoting malignant phenotypes and aggressive tumor behaviors [35]. Therefore, the authors hypothesized that HIF1A overexpression might contribute to poor clinical outcomes by providing a growth advantage under hypoxic stress, accelerating tumor invasion and metastasis.

Subsequently, we explored the mechanism of HIF1A regulating PTC. HIF1A was mainly involved in immune-associated pathways of JAK-STAT, Toll-like receptor, hematopoietic cell lineage, as well as stroma-associated pathways including cell adhesion molecules, focal adhesion, and ECM-receptor interaction pathway. Aberrant activation of the Toll-like receptor signaling pathway leads to NF-κB signaling activation and overexpression of inflammatory cytokines such as IL-6, IL-1β and TNF-α [36]. JAK-STAT signaling mediates almost all immune regulatory processes, including those involved in tumor cell recognition and tumor-driven immune escape [37]. The presence of stimulatory or inhibitory signals governs the innate and adaptive immune activity, controlling effective immune surveillance and facilitating escape. These signals consist of various antigens, foreign ligands, nucleic acids, as well as cytokines and secreted factors that are induced by IFN, TGF-β, and NF-κB pathways regulated by the activation of JAK-STAT [38]. The ECM is a highly dynamic and complex molecular network that surrounds cells in tissues. Focal adhesion is a subcellular structure that provides strong adhesion to the ECM and serves as a scaffold for many integrin-involved signaling pathways [39]. In addition, focal adhesion represents an essential role in tumor cell invasion and cell migration [40]. CIBERSORT analysis also showed that HIF1A was negatively correlated with T cells CD8, NK cells activated, monocytes, and macrophages M2, but positively associated with dendritic cells activated, and T cells regulatory. High expression of T cells CD8 indicates obvious T cell reaction in thyroid cancer patients and its infiltration contributed to better OS. Dendritic cells were increased in thyroid cancer tissue, suppressing the immune response [41]. Macrophages M2 favor the Th2 immune response, promoting tumor progression [42]. Taken together, HIF1A expression exhibited negative correlations with anti-tumor immunity and presented a positive association with tumor-suppressive immunity. In addition to tumor cells and immune cells, stromal cells are also important components in the tumor microenvironment. Cytokines secreted by stromal cells activate signaling pathways regulating immune infiltration and EMT [43]. ECM components provide biochemical and biomechanical cues for cells, which are vital in breast cancer progression and metastasis [44]. The focal adhesion pathway has also been revealed to participate in the EMT process, and its activation can alter cell glycolysis, and induce drug resistance [45,46]. Thus, HIF1A upregulation might lead to a worse prognosis through the activation of these oncogenic pathways.

The comprehensive study is potentially valuable in advancing the understanding of the relationship between HIF1A and PTC, but some limitations still exist. Although we performed the IHC analysis to determine the HIF1A protein expression in tissues of PTC and normal thyroid, the role of HIF1A in tumor metastasis and immune cell infiltration require further study in the future. Second, most of the analyses performed in the study were based on HIF1A mRNA level, and deeper analysis based on protein level would increase the evidence level of the data. These results should be validated in a large-scale population in the future.

In conclusion, the HIF1A expression was increased in PTC tissue, and its expression was positively linked to the LNM stage. HIF1A overexpression could predict high LNM risk and poor DFI, indicating that HIF1A might serve as a reliable biomarker for PTC patients. Moreover, HIF1A might influence survival of PTC patients via regulating immune-as well as stroma-related pathways.

## Disclosure of interest

The authors report no conflict of interest.

## Consent for publication

Not applicable.

## Data sharing statement

The datasets used and/or analyzed during the current study are available from the corresponding author on reasonable request.

## Ethics approval

The study was conducted in accordance with the Declaration of Helsinki, and approved by the Institutional Review Board of the First Hospital of Lanzhou University.

## Funding

This work was supported by the following grants: 10.13039/501100004775Natural Science Foundation of Gansu Province, China [No. 17JR5RA272; No. 22JR5RA923] and 10.13039/100012899Research Fund project of The First Hospital of Lanzhou University [No. Ldyyyn2021-120; No. Ldyyyn2020-98; No. Ldyyyn2021-30].

## Authors' contributions

Yong-xun Zhao and Yang Ze conceived, designed and performed the experiments; Li-bin Ma and Fang Wang analyzed and interpreted the data; Yong Wang and Cheng Xiang contributed reagents, materials, analysis tools or data. All authors wrote and approved the final manuscript.

## References

[1] Singh Ospina N., Iniguez-Ariza N.M., Castro M.R. (2020). Thyroid nodules: diagnostic evaluation based on thyroid cancer risk assessment. BMJ.

[2] Siegel R.L., Miller K.D., Jemal A. (2020). Cancer statistics. Ca - Cancer J. Clin..

[3] Siegel R.L., Miller K.D., Jemal A. (2019). Cancer statistics. Ca - Cancer J. Clin..

[4] Li M. (2022). Identification of transcriptional pattern related to immune cell infiltration with gene Co-expression network in papillary thyroid cancer. Front. Endocrinol..

[5] Schwaiger K. (2019). Occult papillary thyroid cancer presenting as cystic metastasis of the lateral neck: a case report. Medicine (Baltim.).

[6] Vuong H.G. (2018). Prognostic importance of solid variant papillary thyroid carcinoma: a systematic review and meta-analysis. Head Neck.

[7] Wang H.H., Ma J.N., Zhan X.R. (2021). Circular RNA Circ_0067934 attenuates ferroptosis of thyroid cancer cells by miR-545-3p/SLC7A11 signaling. Front. Endocrinol..

[8] Wang L. (2022). Lymph node metastasis of papillary thyroid carcinoma in the context of Hashimoto's thyroiditis. BMC Endocr. Disord..

[9] Rajabi S. (2019). The roles and role-players in thyroid cancer angiogenesis. Endocr. J..

[10] Segura-Villalobos D. (2022). Mast cell-tumor interactions: molecular mechanisms of recruitment, intratumoral communication and potential therapeutic targets for tumor growth. Cells.

[11] Zheng F. (2021). The HIF-1alpha antisense long non-coding RNA drives a positive feedback loop of HIF-1alpha mediated transactivation and glycolysis. Nat. Commun..

[12] Collin L.J. (2021). Hypoxia-inducible factor-1alpha expression and breast cancer recurrence in a Danish population-based case control study. Breast Cancer Res..

[13] Cimmino F. (2019). HIF-1 transcription activity: HIF1A driven response in normoxia and in hypoxia. BMC Med. Genet..

[14] Huang Y., Lin D., Taniguchi C.M. (2017). Hypoxia inducible factor (HIF) in the tumor microenvironment: friend or foe?. Sci. China Life Sci..

[15] Zhang Y. (2021). HIF-1alpha is necessary for activation and tumour-promotion effect of cancer-associated fibroblasts in lung cancer. J. Cell Mol. Med..

[16] Palazon A. (2014). HIF transcription factors, inflammation, and immunity. Immunity.

[17] Feng Y. (2022). ARID1A is a prognostic biomarker and associated with immune infiltrates in hepatocellular carcinoma. Chin. J. Gastroenterol. Hepatol..

[18] Hanzelmann S., Castelo R., Guinney J. (2013). GSVA: gene set variation analysis for microarray and RNA-seq data. BMC Bioinf..

[19] Hsiao Y.W. (2019). Tumor-infiltrating leukocyte composition and prognostic power in hepatitis B- and hepatitis C-related hepatocellular carcinomas. Genes.

[20] Tang X. (2022). Identification of the ferroptosis-related long non-coding RNAs signature to improve the prognosis prediction in papillary renal cell carcinoma. Front Surg.

[21] Huo J., Wu L., Zang Y. (2021). Development and validation of a robust immune-related prognostic signature for gastric cancer. J Immunol Res.

[22] Luo Y. (2018). Model of lymph node metastasis posterior to the right recurrent laryngeal nerve in papillary thyroid carcinoma. Cancer Manag. Res..

[23] Mariniello R.M. (2020). The TUSC2 tumour suppressor inhibits the malignant phenotype of human thyroid cancer cells via SMAC/DIABLO protein. Int. J. Mol. Sci..

[24] Liu X. (2021). Annexin A10 is a novel prognostic biomarker of papillary thyroid cancer. Ir. J. Med. Sci..

[25] Qin X.J. (2021). CXCL10 is a potential biomarker and associated with immune infiltration in human papillary thyroid cancer. Biosci. Rep..

[26] Yang Z. (2021). A new risk factor indicator for papillary thyroid cancer based on immune infiltration. Cell Death Dis..

[27] Zheng J.J. (2020). Hypoxia activates SOX5/wnt/beta-catenin signaling by suppressing MiR-338-3p in gastric cancer. Technol. Cancer Res. Treat..

[28] Grassilli E., Cerrito M.G. (2022). Emerging actionable targets to treat therapy-resistant colorectal cancers. Cancer Drug Resist.

[29] Lu J. (2020). Ginsenoside 20(S)-Rg3 upregulates HIF-1alpha-targeting miR-519a-5p to inhibit the Warburg effect in ovarian cancer cells. Clin. Exp. Pharmacol. Physiol..

[30] Wang J.Z. (2021). The role of the HIF-1alpha/ALYREF/PKM2 axis in glycolysis and tumorigenesis of bladder cancer. Cancer Commun..

[31] Cohen M. (2019). beta-TrCP upregulates HIF-1 in prostate cancer cells. Prostate.

[32] Koperek O. (2013). Expression of hypoxia-inducible factor 1 alpha in papillary thyroid carcinoma is associated with desmoplastic stromal reaction and lymph node metastasis. Virchows Arch..

[33] Liu Y.M. (2016). Expression of HIF-1alpha and HIF-2 alpha correlates to biological and clinical significance in papillary thyroid carcinoma. World J. Surg. Oncol..

[34] Tsai Y.P. (2014). TET1 regulates hypoxia-induced epithelial-mesenchymal transition by acting as a co-activator. Genome Biol..

[35] de Oliveira J.T. (2015). Hypoxia up-regulates galectin-3 in mammary tumor progression and metastasis. PLoS One.

[36] Moradi-Marjaneh R. (2018). Toll like receptor signaling pathway as a potential therapeutic target in colorectal cancer. J. Cell. Physiol..

[37] Owen K.L., Brockwell N.K., Parker B.S. (2019). JAK-STAT signaling: a double-edged sword of immune regulation and cancer progression. Cancers.

[38] Villarino A.V., Kanno Y., O'Shea J.J. (2017). Mechanisms and consequences of Jak-STAT signaling in the immune system. Nat. Immunol..

[39] Burridge K. (2017). Focal adhesions: a personal perspective on a half century of progress. FEBS J..

[40] Shen J. (2018). Hippo component YAP promotes focal adhesion and tumour aggressiveness via transcriptionally activating THBS1/FAK signalling in breast cancer. J. Exp. Clin. Cancer Res..

[41] Joyce J.A., Fearon D.T. (2015). T cell exclusion, immune privilege, and the tumor microenvironment. Science.

[42] Ran T. (2021). LAMB1 is related to the T stage and indicates poor prognosis in gastric cancer. Technol. Cancer Res. Treat..

[43] Slabakova E. (2015). Opposite regulation of MDM2 and MDMX expression in acquisition of mesenchymal phenotype in benign and cancer cells. Oncotarget.

[44] Insua-Rodriguez J., Oskarsson T. (2016). The extracellular matrix in breast cancer. Adv. Drug Deliv. Rev..

[45] Ning Z. (2014). USP22 promotes epithelial-mesenchymal transition via the FAK pathway in pancreatic cancer cells. Oncol. Rep..

[46] Wang S. (2016). Cisplatin suppresses the growth and proliferation of breast and cervical cancer cell lines by inhibiting integrin beta5-mediated glycolysis. Am J Cancer Res.

