# Supplementary material for: HIF1A overexpression predicts the high lymph node metastasis risk and indicates a poor prognosis in papillary thyroid cancer
Source: Heliyon. 2023 Mar 17;9(3):e14714. doi: 10.1016/j.heliyon.2023.e14714 (PMC10040699; doi:10.1016/j.heliyon.2023.e14714)
Supplement: Multimedia component 1 [file mmc1.docx]

| **Supplementary table 1** Patient characteristics | | | |
| --- | --- | --- | --- |
|  | Total (n) | Low HIF1A | High HIF1A |
| Age |  |  |  |
| <55 | 333 (67.0) | 163 (65.7) | 170 (68.3) |
| ≥55 | 164 (33.0) | 85 (34.3) | 79 (31.7) |
| Gender |  |  |  |
| Female | 363 (73.0) | 179 (72.2) | 184 (73.9) |
| Male | 134 (27.0) | 69 (27.8) | 65 (26.1) |
| LNM stage |  |  |  |
| N0 | 224 (45.1) | 130 (52.4) | 94 (37.8) |
| N1 | 225 (45.3) | 90 (36.3) | 135 (54.2) |
| Unknown | 48 (9.6) | 28 (11.3) | 20 (8.0) |
| OS status |  |  |  |
| Alive | 481 (96.8) | 243 (98.0) | 238 (95.6) |
| Dead | 16 (3.2) | 5 (2.0) | 11 (4.4) |
| DSS status |  |  |  |
| Alive | 490 (98.6) | 246 (99.2) | 244 (98.0) |
| Dead | 7 (1.4) | 2 (0.8) | 5 (2.0) |
| DFI status |  |  |  |
| Disease-free | 471 (94.8) | 241 (97.2) | 230 (92.4) |
| Recurrence | 26 (5.2) | 7 (2.8) | 19 (7.6) |
| PFI status |  |  |  |
| Progression-free | 446 (89.7) | 228 (91.9) | 218 (87.6) |
| Progression | 51 (10.3) | 20 (8.1) | 31 (12.4) |
